# Profound Functional Suppression of Tumor-Infiltrating T-Cells in Ovarian Cancer Patients Can Be Reversed Using PD-1-Blocking Antibodies or DARPin® Proteins

**DOI:** 10.1155/2020/7375947

**Published:** 2020-08-04

**Authors:** Emelie Foord, Charlotte Klynning, Esther Schoutrop, Judith M. Förster, Jennifer Krieg, Anette Mörtberg, Mischa R. Müller, Christel Herzog, Dieter Schiegg, Denis Villemagne, Ulrike Fiedler, Dan Snell, Benjamin Kebble, Jonas Mattsson, Victor Levitsky, Michael Uhlin

**Affiliations:** ^1^Department of Clinical Science, Intervention and Technology, Karolinska Institutet, Stockholm, Sweden; ^2^Department of Gynecological Oncology, Karolinska University Hospital, Stockholm, Sweden; ^3^Department of Oncology/Pathology, Karolinska Institutet, Stockholm, Sweden; ^4^Department of Translational Oncology, German Cancer Research Center and National Center for Tumor Diseases, Heidelberg, Germany; ^5^Faculty of Biosciences, Heidelberg University, Heidelberg, Germany; ^6^Molecular Partners AG, Zurich-Schlieren, Switzerland; ^7^Department of Immunology and Transfusion Medicine, Karolinska University Hospital, Stockholm, Sweden; ^8^Division of Medical Oncology and Hematology, Princess Margaret Cancer Centre and University of Toronto, Toronto, ON, Canada; ^9^Department of Applied Physics, Royal Institute of Technology, Stockholm, Sweden

## Abstract

PD-1/PD-L1 blockade has revolutionized the field of immunooncology. Despite the relative success, the response rate to anti-PD-1 therapy requires further improvements. Our aim was to explore the enhancement of T-cell function by using novel PD-1-blocking proteins and compare with clinically approved monoclonal antibodies (mAbs). We isolated T-cells from the ascites and tumor of 17 patients with advanced epithelial ovarian cancer (EOC) and analyzed the effects using the mAbs nivolumab and pembrolizumab and two novel engineered ankyrin repeat proteins (DARPin® proteins). PD-1 blockade with either mAb or DARPin® molecule significantly increased the release of IFN-*γ*, granzyme B, IL-2, and TNF-*α*, demonstrating successful reinvigoration. The monovalent DARPin*®* protein was less effective compared to its bivalent equivalent, demonstrating that bivalency brings an additional benefit to PD-1 blockade. Overall, we found a higher fold increase of lymphokine secretion in response to the PD-1 blockade by tumor-derived T-cells; however, the absolute amounts were significantly lower compared to the release from ascites-derived T-cells. Our results demonstrate that PD-1 blockade can only partially reinvigorate functionally suppressed T-cells from EOC patients. This warrants further investigation preferably in combination with other therapeutics. The study provides an early pilot proof-of-concept for the potential use of DARPin*®* proteins as eligible alternative scaffold proteins to block PD-1.

## 1. Introduction

Many human solid tumors are known to contain tumor-infiltrating lymphocytes (TILs) [1–4]; however, tumor cells are able to avoid elimination through various escape mechanisms including functional suppression of T-cells. Impaired TIL functionality is characterized by defective cytotoxic activity, diminished cytokine secretion, and failure to proliferate in response to stimulation [5]. This exhausted state is associated with the expression of several surface co-inhibitory receptors, which can be expressed on TILs in various combinations and suppress cell functions by interacting with ligands on tumor cells and other suppressive cell types [5–8].

Blockade of programmed cell death protein-1 (PD-1) acts through interference of one of the major co-inhibitory receptor/ligand interactions on TILs. The use of the PD-1/PD-L1 blockade has been clinically successful in a large number of advanced stage tumors including melanoma and non-small cell lung cancer [9–11]. There are currently two monoclonal antibodies (mAbs) targeting the PD-1 receptor, nivolumab and pembrolizumab, approved by the US Food and Drug Administration. Despite the clinical success, there are still many aspects of this strategy requiring additional optimization including (a) identification of benefitting patient groups, (b) finding relevant combination therapies to improve clinical efficacy, (c) optimizing treatment schedules, (d) identification of biomarkers, (e) limiting adverse events, and (f) managing acquired resistance [12, 13].

DARPin® proteins are small high-affinity ankyrin repeat proteins which are easily designed and engineered. They consist of varying numbers of modules and range in sizes from 14 to 21 kDa, making them substantially smaller than traditional monoclonal IgG antibodies (~150-200 kDa) and F(ab') fragments (~60 kDa) [14]. DARPin® constructs can be designed as multivalent and multispecific. From a therapeutic point of view, DARPin® molecules have several advantageous properties including high stability and solubility, high potency, simple and flexible design, and rapid manufacturing in *Escherichia coli* at low cost [14]. They can be manufactured with different attached functions, such as the ability to bind human serum albumin, prolonging the half-life of the construct. Furthermore, the absence of an Fc-receptor-engaging part excludes potentially unwanted effector functions such as cellular and complement-mediated cytolytic activities. This makes DARPin*®* proteins good candidates in therapies targeting T-cells, as is the case with PD-1-targeted antagonists.

In the current study, the aim was to explore novel DARPin® proteins as PD-1-targeting alternative scaffold proteins and compare the effects with the two available mAbs. We assessed the functionality of T-cells isolated from the ascites and tumor of patients with advanced epithelial ovarian cancer (EOC). EOC was chosen as the model system due to the identified strong favorable prognostic value of infiltrating T-cells [15, 16] and relatively limited/modest response rate to PD-1 antagonizing treatment in the clinics; smaller clinical trials using PD-1/PD-L1-directed mAbs have shown approximately 0-15% overall response rate [17–21]. The use of patient-derived material allowed us to explore whether conventional and novel PD-1 blockade *ex vivo* could improve T-cell response in EOC, as assessed by the release of important effector molecules. Our findings show an increased functionality of suppressed T-cells from the ascites and tumor in response to PD-1 blockade. The reinvigoration of T-cells was comparable using conventional PD-1-blocking mAbs and a bivalent DARPin® protein, highlighting the potential in immunotherapy of cancer.

## 2. Material and Methods

### 2.1. Patient Material

Peripheral blood (*n* = 12), ascites (*n* = 16), and metastatic omental tumor tissue (*n* = 8) were collected from 17 patients undergoing primary surgery for advanced EOC at Karolinska University Hospital (Solna, Sweden) (Table 1). Written informed consent was obtained from all patients at the Women's Health Clinic (Karolinska University Hospital, Solna, Sweden). No patient received chemotherapy prior to surgery. Peripheral blood mononuclear cells (PBMCs) from healthy anonymous blood donors were used for characterization of PD-1 and effector function potential (*n* = 10) as well as mixed lymphocyte reactions (MLRs) (*n* = 3). The study was carried out in accordance with the Helsinki Declaration and was approved by the Regional Ethical Review Board of Stockholm, Sweden (2013/2161-31/2, 2016/1136-32, and 2016/1631-32).

### 2.2. Isolation of Mononuclear Cells and De Novo PD-1 Expression

Patient material was collected and processed as previously published [22]. Briefly, the tumor was mechanically dissociated, filtered, and washed in PBS. Mononuclear cells from all sample types were isolated by density gradient centrifugation with Lymphoprep (1.077 g/cm^2^, Fresenius Kabi) and characterized for a large number of markers as previously published, including the *de novo* expression of PD-1 [22]. Presented data on PD-1 expression reflects the samples used for the PD-1-blocking experiments, and detailed information on the staining procedure can be found in our previous publication [22]. Cells used for later activation and PD-1 blocking were cryopreserved in a HyClone RPMI-1640 medium (GE Healthcare Life Sciences) with 10% heat-inactivated AB serum (Karolinska University Hospital) and 10% CryoSure dimethyl sulfoxide (WAK-Chemie Medical GmbH) and stored at -192°C until use.

### 2.3. PD-1 Targeting Reagents

Monoclonal antibodies nivolumab (Opdivo**®**, Bristol Myers Squibb) and pembrolizumab (Keytruda**®**, Merck & Co., Inc.) along with two DARPin® proteins (DARPin-1 and DARPin-2) were used for PD-1 blocking. DARPin® proteins were developed and provided by Molecular Partners AG. Human IgG4*λ* (Sigma-Aldrich) and a negative control DARPin® protein (NCD) lacking PD-1-binding domains were used as negative controls. Sharing of DARPin® proteins may be subject to a Material Transfer Agreement or limited by the filing of patent applications.

### 2.4. Binding to Human PD-1

Determination of PD-1 binding was performed with an in-house transfected stable PD-1-expressing human HEK293 cell line. A titration of mAb or DARPin® molecule was incubated with 1 × 10^5^ HEK293 cells for 30 min at 4°C. After washing, binding of mAbs was detected by Alexa Fluor 488-conjugated goat anti-human IgG (H+L) (LubioScience) and binding of DARPin® molecules was detected by Alexa Fluor 488-conjugated mouse anti-Penta-His (QIAGEN). After 30 min incubation at 4°C, cells were washed and resuspended in a Cytofix fixation buffer (BD Biosciences). After fixation, 5,000 cells were counterstained by 5 *μ*M DRAQ5 (Abcam) for 15 min at RT. Median of mean fluorescence intensities (MFI) of Alexa Fluor 488 binding on far-red counterstained cells was measured by Mirrorball laser scanning imaging cytometry using Cellista software (SPT Labtech). MFI of the remaining fixed cells were measured by flow cytometry on a FACS Canto II using BD FACSDiva Software v.7.0 (BD Biosciences).

### 2.5. Reporter Cell Assay

PD-1/PD-L1 blockade bioassay (Promega, *n* = 2) was performed as described in the manufacturer's protocol. In brief, PD-1+ effector Jurkat T-cells were incubated with PD-L1+ aAPC/CHO-K1 cells in the presence of dose titrations of nivolumab or DARPin® molecules. After 6 h incubation at 37°C, Bio-Glo™ reagent was added and the luminescence was quantified using a fluorimeter (TECAN Infinite M1000 PRO).

### 2.6. Mixed Lymphocyte Reactions

Monocytes and CD4+ T-cells were isolated from PBMCs using the Human Monocyte Isolation Kit II and Human CD4+ T Cell Isolation Kit as instructed by the manufacturer (Miltenyi Biotec). Dendritic cells (DCs) were generated by culturing monocytes *in vitro* for 7 days with 500 U/mL interleukin-4 (IL-4, Miltenyi Biotec) and 1,000 U/mL granulocyte-macrophage colony-stimulating factor (GM-CSF, Miltenyi Biotec). Purified CD4+ T-cells (1 × 10^5^) and generated allogeneic DCs (1 × 10^4^) were co-cultured with or without dose titrations of DARPin® molecule or nivolumab added at the initiation of the assay. After 6 days, IFN-*γ* secretion in culture supernatants was analyzed using the Human IFN-*γ* standard ABTS enzyme-linked immunosorbent assay (ELISA) development kit (PeproTech). The results were assessed using a TECAN Infinite M1000 PRO plate reader at 405 nm with wavelength correction set at 650 nm.

### 2.7. Effects of PD-1 Blockade on Patient-Derived T-Cells by ELISA and Luminex

For activation and PD-1 blocking of patient-derived T-cells, thawed mononuclear cells were counted and plated at 1 × 10^6^ lymphocytes/mL in complete RPMI-1640 medium (10% AB serum and 1% penicillin-streptomycin, GE Healthcare Life Sciences). No further purification of T-cells was performed in order to maintain a mixture of cells in the cultures. Agonistic *α*-CD3 (OKT-3, BioLegend, 50 ng/mL) was added together with PD-1-directed reagents or negative controls (100 nM or dose titrations). After 48 h at 37°C with 5% CO_2_, supernatants were collected and stored at -80°C. IFN-*γ* was measured in thawed supernatants using the Human IFN-*γ* ELISA PRO Kit (Mabtech) as instructed by the manufacturer. The results were read using a VMax kinetic microplate reader (Molecular Devices) at 450 nm with wavelength correction set at 630 nm.

The secreted IFN-*γ* was assumed to be originating from T-cells as *α*-CD3 only stimulates this subset. Fluorescent bead-based multiplex immunoassay (Luminex) was also performed on a more limited number of supernatants using the MILLIPLEX MAP Human CD8+ T-Cell Magnetic Bead Panel Kit 96-well assay (HCD8MAG-15K, Merck Group) with IFN-*γ*, granzyme B, IL-2, TNF-*α*, IL-10, and soluble 4-1BB (s4-1BB)/sCD137 or IL-6. IL-6 was initially analyzed but was replaced by s4-1BB as anti-PD-1 reagents did not appear to cause changes in release. Some samples reached the upper detection limit and were excluded from further analysis. The procedure was performed as instructed by the manufacturer and previously described [23] and was analyzed on a Luminex 200 with xPONENT 4.2 (Luminex Corporation). Calibration curves and concentrations were calculated with SoftMax Pro v.6.2.2 software (Molecular Devices).

### 2.8. Extracellular Staining Postactivation by Flow Cytometry and Normalization of Data

Cellular composition of the cultured cells was analyzed with murine-derived anti-human antibodies from BD Biosciences (Table S1). Cells were stained with antibodies in PBS at 4°C for 20 min followed by washing and viability staining using 7AAD according to the manufacturer (BD Biosciences). Cells were acquired on a BD FACSCanto using BD FACSDiva software v.7.0, and the data was analyzed in FlowJo v.10 (BD Biosciences). Percentage of T-cells (gated on singlets/living cells/lymphocytes) in unstimulated samples was analyzed and used for normalization of the lymphokine data generated by ELISA and Luminex.

Concentrations of cytokine and effector molecule secretion post-activation were normalized to reflect 500,000 T-cells. Relative fold induction was calculated as a ratio of the response observed when only adding *α*-CD3 and the condition including anti-PD-1 construct or negative control.

### 2.9. Data Analysis and Statistics

Prism 7 (GraphPad Software Inc.) was used for calculations, statistics, and graphing. The non-parametric Wilcoxon signed rank test and Mann-Whitney *U*-test were used for statistical comparisons. Median values were used in Results. Non-parametric Spearman correlation was used and plotted together with nonlinear regression. Differences were considered statistically significant if ^∗^*p* < 0.05, ^∗∗^*p* < 0.01, or ^∗∗∗^*p* < 0.001.

## 3. Results

### 3.1. Pronounced Expression of PD-1 on Ascites-Derived and Tumor-Derived T-Cells

**Figure 1:**
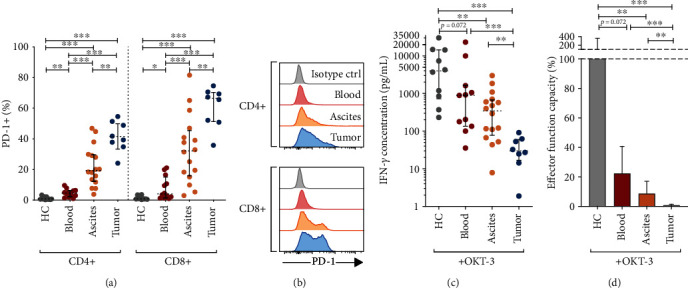
PD-1 expression and effector function capacity of T-cells isolated from healthy controls (HC) and epithelial ovarian cancer (EOC) patients. (a) Proportion of PD-1+ (%) CD4+ and CD8+ T-cells isolated from the peripheral blood of HC (*n* = 10) and the blood (*n* = 12), ascites (*n* = 16), and tumor (*n* = 8) of EOC patients. Gating strategy included singlets, followed by viable cells (being 7AAD-), lymphocyte gate (by forward/side scatter), and T-cells (CD3+), followed by expression of CD4+ and CD8+ and lastly by expression of PD-1 on these subsets. (b) Representative histograms of PD-1 expression on CD4+ and CD8+ T-cells from one patient. Cells from the tumor were used for the isotype control. (c) Absolute concentrations of IFN-*γ* (normalized to reflect per 500,000 T-cells) after stimulation with *α*-CD3 (OKT-3) for 48 h. (d) The median release of IFN-*γ* in HC after stimulation with *α*-CD3 was set to 100% and defined full response (effector function capacity). The release measured in the samples from EOC patient blood, ascites, and tumor was compared to the release observed in HC blood samples. Median values with interquartile ranges are presented. IFN-*γ* was measured using ELISA, and the data was normalized to reflect the same cell number (per 500,000 T-cells). Unpaired Mann-Whitney was performed as statistical analysis. Significance levels were set to ^∗^*p* < 0.05, ^∗∗^*p* < 0.01, and ^∗∗∗^*p* < 0.001.

We isolated lymphocytes from peripheral blood, ascites, and metastatic omental tumor tissue of advanced EOC patients undergoing tumor-debulking surgery (Table 1). Using flow cytometry, PD-1 expression on T-cells was initially investigated. As expected, a majority of tumor-infiltrating CD8+ T-cells and a large proportion of ascites-derived T-cells expressed PD-1 (Figures 1(a) and 1(b)). A median of 66.5% of CD8+ TILs and 32.2% of CD8+ ascites-derived T-cells expressed PD-1, compared to T-cells isolated from the blood of healthy controls (HC, median 0.8%) and patient blood (4.0%) (Figure 1(a)). The proportion of CD4+ T-cells expressing PD-1 was generally lower compared to CD8+ T-cells but still significantly elevated in the tumor and ascites (40.6% of tumor-derived and 19.3% of ascites-derived CD4+ T-cells) compared to healthy individuals and patient blood (0.7% and 4.6%, respectively) (Figures 1(a) and 1(b)).

### 3.2. Reduced T-Cell Effector Function Capacity in EOC Patients Compared to Healthy Individuals

Next, isolated mononuclear cell fractions from blood, ascites, and tumor were incubated with *α*-CD3 for 48 h, activating T-cells non-specifically. The release of IFN-*γ* was measured as a readout of effector function capacity. The results showed tumor-derived T-cells to have a significantly reduced capacity to produce IFN-*γ* compared to T-cells isolated from ascites (*p* = 0.001) and blood of patients as well as HC (*p* < 0.001 for both) (Figure 1(c)). Ascites-derived T-cells also had decreased IFN-*γ* secretion compared to blood of HC (*p* = 0.001), but unexpectedly, no significant difference was found compared to the blood-derived T-cells of the patients. Instead, there was a trend for patient blood T-cells to have reduced IFN-*γ* secretion compared to HC (*p* = 0.072) (Figure 1(c)).

To compare the functionality of T-cells isolated from different sites of EOC patients, the median amount of IFN-*γ* produced by T-cells from the blood of healthy donors was defined as 100% response. Relative to this value, the median response by T-cells from the blood or ascites of EOC patients reached only 22.3% (4.5-fold decrease) and 8.7% (11.5-fold decrease), respectively, compared to T-cell activation from HC (Figure 1(d)). Tumor-derived T-cells had less than 1% relative response rate compared to HC blood T-cells (Figure 1(d)). These findings were based on normalized data, reflecting the same number of T-cells (per 500,000 T-cells), and demonstrate compromised T-cell functionality in EOC patients, in particular at tumor sites. Expression of one of the PD-1 ligands, PD-L1, was assessed in six paired samples of the ascites and tumor, and the results confirmed PD-L1 expression on CD45- cells (which includes tumor cells) in the *ex vivo* cell fractions (both unstimulated and stimulated conditions) (Supplementary Figure 1).

The reduced functional capacity of T-cells isolated from the ascites and tumor samples was hypothesized to be associated with the co-inhibitory receptor expression. Except for identifying a negative correlation between the release of IFN-*γ* in ascites and PD-1 expression among ascites-derived CD4+ T-cells (*r* = −0.51, *p* = 0.044) (Supplementary Figure 2), no other correlations between PD-1 expression on CD4+ or CD8+ T-cells and IFN-*γ* release were found in the different sample types (blood, ascites, and tumor). To further investigate the reduced functional capacity, we wanted to assess whether the dysfunction could be reversed using PD-1 blockade with conventional and novel constructs.

### 3.3. Binding and Titration of PD-1-Targeting Reagents

**Figure 2:**
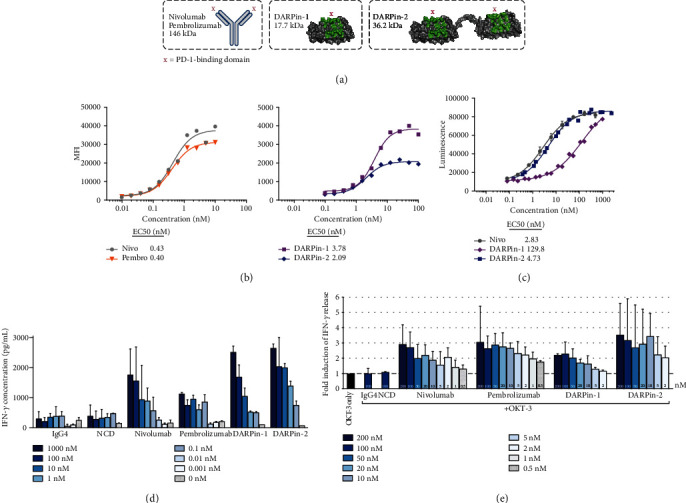
Overview of PD-1-blocking monoclonal antibodies and DARPin® proteins. (a) Schematic of the PD-1-binding reagents: two conventional monoclonal antibodies (mAbs) of IgG4 subtype (nivolumab and pembrolizumab) and two DARPin® proteins with monovalent (DARPin-1) or bivalent (DARPin-2) PD-1 binding. (b) Assessment of PD-1 binding using a titration of PD-1-targeting reagents with a PD-1-expressing cell line (transfected HEK293). PD-1 binding was measured by mean fluorescence intensity (MFI) using flow cytometry. Half-effective concentrations (EC50 values) are presented for each PD-1-binding reagent—nivolumab (nivo) in circles, pembrolizumab (pembro) in reversed triangles, DARPin-1 in squares, and DARPin-2 in diamonds. (c) PD-1/PD-L1 blockade bioassay (Promega) (*n* = 2) was performed using PD-1+ effector T-cells and PD-L1-expressing aAPC/CHO-K1 cells in the presence of dose titrations of PD-1-targeting reagents. Increased luminescence indicates recovered T-cell activation as PD-1 engagement with PD-L1 interferes with the T-cell receptor-mediated transcription of the reporter gene luciferase. (d) Mixed lymphocyte reaction was performed using CD4+ T-cells and allogeneic dendritic cells generated from healthy donors in the presence of PD-1-targeting mAbs (nivolumab, *n* = 3; pembrolizumab, *n* = 1), DARPin® proteins (*n* = 2 for both), or corresponding controls (human IgG4 and negative control DARPin® protein, NCD, respectively, *n* = 2 for both). The levels of IFN-*γ* in the supernatants were analyzed using ELISA, and results are presented for indicated concentrations. (e) Fold induction of IFN-*γ* release from T-cells isolated from the ascites or tumor from ovarian cancer patients (*n* = 7) after 48 h incubation with *α*-CD3 (OKT-3) and different concentrations of PD-1-targeting mAbs (*n* = 8), DARPin® proteins (*n* = 4), or corresponding controls (IgG4 and NCD, *n* = 8 and *n* = 4, respectively, both used at 100 nM). The response was normalized based on the response with only *α*-CD3, which was set to 1 (dashed line). The results are presented as a median with interquartile range, and concentrations are indicated in the figure.

A schematic of the used PD-1-directed constructs is shown in Figure 2(a). The bivalent DARPin® protein (DARPin-2) is a duplicate of the monovalent construct (DARPin-1) with a linker connecting the two domains. Both constructs have an N-terminal MRGS(H)6 tag and are considerably smaller (DARPin-1: 17.7 kDa, DARPin-2: 36.2 kDa) compared to the monoclonal antibodies nivolumab and pembrolizumab (both 146 kDa). We assessed the binding capacity by incubating PD-1+ HEK293 cells with increasing doses of the reagents (0.1-10 nM for mAbs and 0.1-100 nM for DARPin® proteins) followed by fluorescence labeling of the anti-PD-1-proteins (anti-IgG and anti-Penta-His) (Figure 2(b)). Calculations of half-effective concentrations (EC50) showed comparable activities of both mAbs (0.43 nM and 0.40 nM for nivolumab and pembrolizumab, respectively) while the results for the DARPin® proteins showed approximately a 5-fold difference in the binding to PD-1+ cells with DARPin-2 and a 10-fold difference with DARPin-1 compared to mAbs, with EC50 values of 2.1 and 3.8 nM, respectively (Figure 2(b)). The results were confirmed using Mirrorball laser scanning imaging cytometry (*n* = 1 for all reagents, Supplementary Figure 3).

The efficacy of the PD-1 blockade was assessed using a reporter cell assay. In this assay, PD-1+ Jurkat T-cells carrying a luciferase-encoding reporter gene regulated by the T-cell receptor-driven NFAT signaling pathway were co-cultured with PD-L1+ TCR-activating aAPC/CHO-K1 cells. In this experimental system, PD-1/PD-L1 interactions inhibit transcription of the reporter gene while successful blocking of the interaction results in activation of the NFAT pathway, expression of the reporter gene, and increased luminescent signal. Using this approach to study the efficacy of three of the PD-1 antagonists, all were found to induce the expression of luciferase in a dose-dependent manner (Figure 2(c)). The bivalent DARPin-2 and nivolumab showed comparable potency (EC50 4.73 nM and 2.83 nM, respectively, a 1.6-fold difference) (Figure 2(c)). In contrast, the monovalent DARPin-1 showed greatly decreased potency compared with DARPin-2 (EC50 129.8 nM, a 27-fold difference) (Figure 2(c)).

Next, we assessed the capacity of PD-1-blocking constructs to induce secretion of IFN-*γ*. To this end, PD-1-blocking constructs were titrated in MLR assays with cells from healthy donors (Figure 2(d)) and *α*-CD3-based activation of ascites- and tumor-derived T-cells (Figure 2(e)). The two methods showed comparable results with overall similar potency of the bivalent DARPin-2, nivolumab, and pembrolizumab. However, in line with the findings from the reporter cell assay, the monovalent DARPin-1 was not as potent as its bivalent equivalent (Figures 2(d) and 2(e)). Cell viability was not affected by the addition of PD-1-targeting reagents (data not shown).

### 3.4. Enhanced IFN-*γ* Release by Both Anti-PD-1 mAbs and DARPin® Proteins

**Figure 3:**
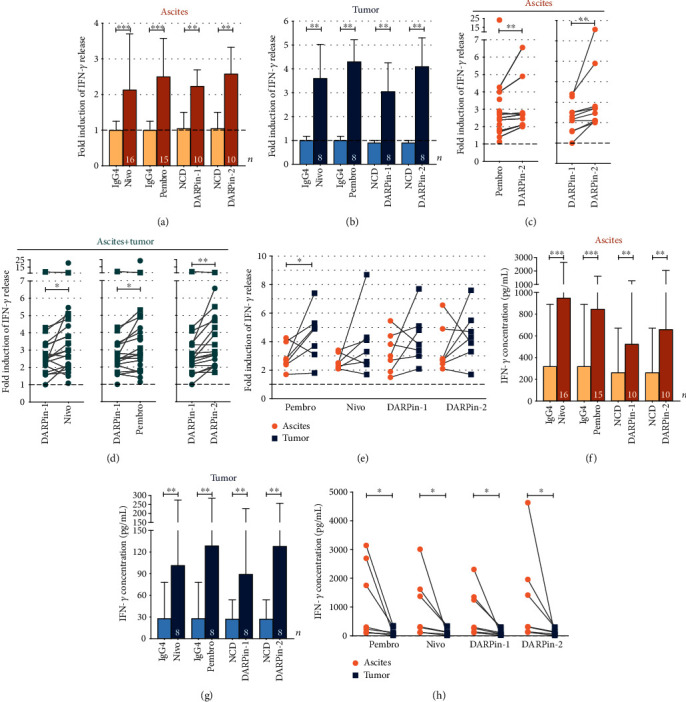
Secretion of IFN-*γ* by T-cells in the presence of *α*-CD3 and PD-1-targeting reagents. T-cells isolated from the ascites (*n* = 16) and tumor (*n* = 8) were activated and cultured with *α*-CD3 (OKT-3) and 100 nM of PD-1-directed reagents (nivolumab, Nivo; pembrolizumab, Pembro; DARPin-1; and DARPin-2) or controls (IgG4 and negative control DARPin® protein, NCD) for 48 h. (a) The relative fold increase of IFN-*γ* when adding PD-1 blockers or control was compared to the release caused by *α*-CD3 alone (represented by a dashed line set to 1). The results are presented for T-cells isolated from the ascites (circles) or (b) tumor (squares) separately. The number of samples is indicated in each bar (*n*). Corresponding controls for anti-PD-1 reagents are presented next to each anti-PD-1. (c) Significant differences among PD-1 blockers in ascites samples are presented separately and also (d) grouped together with tumor samples. Lines represent paired comparisons in which presented reagents have been assessed in parallel (in the same sample). (e) Comparing fold increase in paired samples of the ascites and tumor from the same patient (*n* = 7). Absolute concentrations of IFN-*γ* released by T-cells isolated from (f) the ascites or (g) the tumor. (h) Comparing absolute IFN-*γ* concentrations in paired samples of the ascites and tumor (*n* = 7). All data have been normalized to reflect the same number of T-cells (per 500,000 T-cells). Wilcoxon signed rank test was used, and median values and interquartile ranges are plotted. Significance levels were set to ^∗^*p* < 0.05, ^∗∗^*p* < 0.01, and ^∗∗∗^*p* < 0.001.

We continued to investigate the reinvigoration of T-cell functionality using 100 nM of all PD-1-blocking reagents, which appeared to have a close to maximal effect on IFN-*γ* release (Figures 2(d) and 2(e)). Secretion of IFN-*γ* by T-cells from both ascites and tumor samples upon addition of *α*-CD3 and PD-1-directed constructs was significantly increased compared to corresponding controls (Figures 3(a) and 3(b)). Among ascites-derived T-cells, median-fold IFN-*γ* induction compared to *α*-CD3 alone ranged from a median 2.1-fold by nivolumab to 2.6-fold by DARPin-2 (Figure 3(a)). Paired statistical analysis showed that DARPin-2 induced a significantly increased response compared to pembrolizumab (*p* = 0.016) and DARPin-1 (*p* = 0.004) (Figure 3(c)). Nivolumab induced comparable effects when compared to the other anti-PD-1 reagents when analyzing the response from ascites-derived T-cells (*p* = ns, data not shown).

All PD-1 reagents induced similar median-fold induction of IFN-*γ* (range 3.0-4.3-fold) among tumor-derived T-cells (Figure 3(b)). However, grouping ascites and tumor samples together showed that DARPin-1 induced a lower effect compared to the three other PD-1-targeting reagents (Figure 3(d)). Generally, it appeared that the relative increase of IFN-*γ* was larger among tumor-derived rather than ascites-derived T-cells (Figures 3(a) and 3(b)). To investigate this further, the response in seven paired ascites and tumor samples was compared (Figure 3(e)). The relative response was significantly increased after exposure to pembrolizumab among tumor-derived T-cells compared to ascites-derived T-cells from the same patient (*p* = 0.047, median 4.9-fold compared to 2.7-fold increase, respectively) (Figure 3(e)). Despite similar trends, no statistical significance could be determined for the other PD-1-blocking reagents.

Interestingly, evaluating normalized absolute concentrations revealed striking differences in the amounts of released IFN-*γ* depending on the origin of the T-cells (Figures 3(f) and 3(g)). Similar to earlier findings (Figure 1(c)), levels of secreted IFN-*γ* were approximately 10x lower in tumor samples compared to ascites (Figures 3(f) and 3(g)). Despite increased release of IFN-*γ* with addition of anti-PD-1 reagents, the absolute concentrations in the supernatants of stimulated tumor-derived T-cells did not even reach the baseline levels observed when only adding *α*-CD3 to ascites-derived T-cells (Figures 3(f) and 3(g)). The paired samples of ascites and tumor (*n* = 7) consistently showed decreased IFN-*γ* concentrations in tumor samples compared to ascites, suggesting a more severe dysfunction among the tumor-derived T-cells (*p* = 0.0156 for all) (Figure 3(h)).

### 3.5. Predicting Response Based on PD-1 Expression

Due to the large range in response observed among EOC patient samples, we explored whether the PD-1-blocking-induced IFN-*γ* response was correlated with the expression of PD-1. For both DARPin® constructs, there was a positive correlation between relative IFN-*γ* response and PD-1 expression among CD8+ T-cells (*p* = 0.03 for DARPin-1 and *p* = 0.048 for DARPin-2) (Supplementary Figure 4). Beyond these findings, no apparent correlation between PD-1 expression and response was found (Supplementary Figure 4).

### 3.6. Secretion of Granzyme B, IL-2, TNF-*α*, and IL-10 Is Also Increased upon PD-1 Blockade

**Figure 4:**
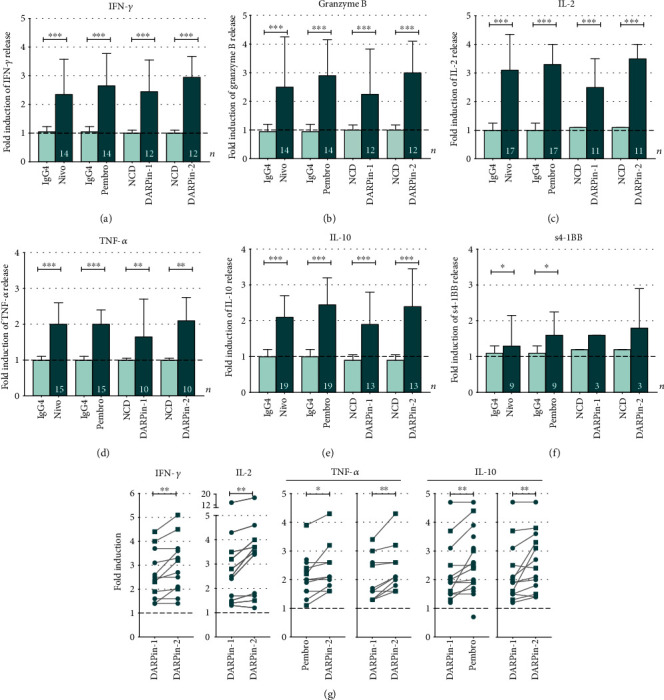
Fold induction of secreted soluble factors from T-cells isolated in the presence of *α*-CD3 and 100 nM of PD-1-directed reagents. T-cells from the ascites (*n* = 14) and tumor (*n* = 5) were activated and cultured with *α*-CD3 (OKT-3) and 100 nM of PD-1-directed reagents or controls for 48 h. Results were generated using a multiplex immunoassay (Luminex) for (a) IFN-*γ*, (b) granzyme B, (c) IL-2, (d) TNF-*α*, (e) IL-10, and (f) soluble 4-1BB (s4-1BB)/sCD137 in samples of ascites and tumor (presented together). Fold induction was calculated based on the release when adding only *α*-CD3 with no presence of anti-PD-1 reagent or control (represented by a dashed line at 1). Several ascites samples reached the upper detection limit in all conditions (including *α*-CD3 alone) and were excluded for several cytokines. The total number of samples evaluated is presented in each bar (*n*). (g) The response by different anti-PD-1 reagents was compared with each other, and significant findings are presented. Although data from the ascites and tumor is pooled together, ascites samples are presented as circles and tumor samples as squares. Lines represent paired comparisons in which presented reagents have been assessed in parallel (in the same sample). Wilcoxon signed rank test was used for all statistical comparisons. Median values and interquartile ranges are plotted. Significance levels were set to ^∗^*p* < 0.05, ^∗∗^*p* < 0.01, and ^∗∗∗^*p* < 0.001.

To further explore the changes in functionality induced by PD-1 blockade, the release of additional important effector molecules was analyzed. Luminex results confirmed the observed increase in release of IFN-*γ* as shown earlier by ELISA (Figure 4(a)). Comparing the results obtained for 16 samples by the two different methods showed a highly significant correlation between the methods (*r* = 0.98, *p* < 0.001 using Spearman correlation; data not shown).

Due to low sample numbers, soluble factor readouts by Luminex assay in the ascites (*n* = 14) and tumor (*n* = 5) were analyzed together, independently of sample site (Figure 4). Levels of secreted granzyme B, IL-2, TNF-*α*, and IL-10 were all significantly increased in response to the combined *α*-CD3 and PD-1 blockade compared to only *α*-CD3 (in general, a 2-3-fold increase) (Figures 4(b)–4(e)). IL-6 and soluble 4-1BB (s-41BB)(s4-1BB) were analyzed for a more limited number of samples, and no difference in the secretion of IL-6 was identified upon addition of PD-1 blockers (Supplementary Figure 5). However, the anti-PD-1 mAbs induced a significant increase of s4-1BB compared to *α*-CD3 alone (Figure 4(f)) and negative control (median 1.3-fold and *p* = 0.047 for nivolumab and 1.6-fold and *p* = 0.016 for pembrolizumab). The DARPin® proteins showed a similar trend; however, they did not yield a statistically significant difference due to limited sample numbers (Figure 4(f)).

Next, the response by different PD-1 blockers was compared with each other in a paired manner (Figure 4(g)). The two mABs showed comparable responses for all analyzed soluble factors. Also, no differences were found between nivolumab and any of the DARPin® proteins. Most striking was the significant difference between the two DARPin® constructs. Monovalent DARPin-1 showed inferior capacity to promote release of IFN-*γ*, IL-2, IL-10, and TNF-*α* compared with the bivalent DARPin-2 (*p* = 0.008, *p* = 0.006, *p* = 0.005, and *p* = 0.004, respectively) (Figure 4(g)). DARPin-1 also had a lower effect on IL-10 secretion compared to pembrolizumab (*p* = 0.008) (Figure 4(g)). Importantly, DARPin-2 induced a significantly increased release of TNF-*α* compared to pembrolizumab (*p* = 0.016).

To summarize, the release of lymphokines was similar for the majority of the PD-1-targeting reagents; however, the monovalent DARPin-1 construct appeared to be the least potent reagent at inducing an increased functional response.

## 4. Discussion

The use of PD-1 blockade has revolutionized cancer treatment. However, significant work remains to be done in order to increase the number of responders, improve patient outcome, and extend clinical benefit to more indications. In the current study, we evaluated the capacity of the clinically approved PD-1-specific mAbs nivolumab and pembrolizumab and novel DARPin® constructs as alternative scaffold proteins to restore dysfunctional T-cells from EOC patients.

We [22] and others have previously identified abundant expression of co-inhibitory receptors on ascites- and tumor-derived T-cells of EOC patients. Checkpoint blockade is therefore an attractive approach towards inducing functional tumor surveillance in this cancer type. To this end, after confirming a pronounced dysfunction among ascites- and tumor-derived T-cells (Figure 1), we explored the use of PD-1 blockade (Figures 2–4). By inhibiting the binding of PD-1 to its ligands PD-L1/PD-L2, expressed on various cell subsets including tumor cells and other cell types in the *ex vivo* cultures, the functionality of T-cells was found to increase, as assessed by the release of several important effector molecules (Figures 3 and 4). When comparing the different PD-1 antagonists, we first found that the two PD-1 mAbs exhibit comparable biological activity (Figures 2–4). This was expected as nivolumab and pembrolizumab, despite some differences in affinity, pharmacology, and administration schedule, have been shown to be very similar in clinical efficacy and toxicity profile [24, 25]. Importantly, these data strongly support the biological relevance of our *ex vivo* assay despite interindividual differences between the analyzed tumor samples such as tumor microenvironment, phenotypic imprints and overall cellular content (regarding the presence of tumor cells, antigen-presenting cells, CD4/CD8 ratio, *etc*.).

Secondly, the results highlighted the potential of using PD-1-targeting alternative protein scaffolds which can match the biological activity of anti-PD-1 mAbs, as demonstrated by the activity of the bivalent DARPin-2 molecule (Figures 2–4). The DARPin® protein technology provides a novel platform in healthcare and has proven to be clinically successful with the development of an anti-vascularr endothelial growth factor (VEGF) DARPin® protein drug candidate for the treatment of macular degeneration [26]. Phase II and III study results have shown this DARPin® protein (Abicipar) to have a safe and efficacious profile in patients [27]. In the context of checkpoint blockade, the overall comparable responses between mAbs and the bivalent DARPin® protein support further investigation of DARPin® protein-based immunotherapeutics. The use of DARPin® proteins might add additional pharmacodynamic benefits such as higher stability and increased tumor penetration [28, 29]. In our study, the bivalent DARPin® protein induced improved IFN-*γ* and TNF-*α* response in some instances compared to pembrolizumab (Figure 3). However, monovalent DARPin-1 was significantly less efficient compared to the other PD-1-blocking reagents (Figures 2–4) suggesting that in this context, bivalency is necessary for optimal PD-1-blocking capacity. This study is considered as a pilot study in the investigation of DARPin® proteins in checkpoint blockade. Nonetheless, the study provides a proof-of-concept and foundation from which further research can continue.

A major obstacle observed in the current study is the overall limited magnitude of response, regardless of which PD-1 blocker construct was used. In line with earlier studies [30], the identified profound state of functional suppression of tumor-derived T-cells was expected. Despite the fact that lymphokine secretion by T-cells increases several fold upon PD-1 blockade, the response, particularly by tumor-derived T-cells, remained exceedingly low in absolute values (Figure 3). Our data thus show that PD-1 blockade most likely is insufficient to reconstitute full functional activity of tumor-derived T-cells of patients with EOC. This may help to explain the fairly limited/modest clinical efficacy of anti-PD-1 treatment in EOC [17–21]. However, many factors are known and hypothesized to influence the response rate. Recent research indicates that an exhausted state of T-cells might be irreversible due to stable epigenetic changes, limiting the effect of checkpoint blockade in general [31, 32]. However, the long-term response observed for a proportion of patients undergoing PD-1 blockade appears to contradict these findings, but there might be alternative explanations such as reinvigoration of certain T-cell subsets [33, 34], recruitment of new T-cells into the tumor site [35], or even differences in microbiota [36]. A limitation of the current study is the lacking knowledge on antigen specificity of the reinvigorated T-cells and whether these are relevant for tumor surveillance, *i.e*, recognizing tumor cells. As recently shown by Scheper *et al*, only a minority of infiltrating T-cells are able to recognize autologous tumor cells [37], and this might be an important underlying factor behind the varying response rate in clinical trials along with other factors such as mutational burden [38, 39] and the factors previously discussed. These aspects were not investigated and should be addressed in the continued investigation of PD-1 blockade in EOC and with the use of DARPin® proteins specifically. Nevertheless, our data show that profound functional dysfunction affects immune cells present in the EOC microenvironment regardless of their antigen specificity.

Despite the obstacles influencing T-cell functionality, our approach of using *ex vivo*-derived TILs may allow a straightforward simple investigation of the effect on functional activity by different combinations of immunomodulatory compounds targeting co-inhibitory, co-stimulatory, and lymphokine receptors in intratumoral T-cells, thereby fostering accelerated development of new therapeutic regiments. Combination treatment is currently under extensive investigation [40], and new combination strategies could also help to prevent acquired adaptive resistance with, for example, compensatory upregulation of other co-inhibitory receptors [41, 42]. Here, another benefit with the DARPin® protein technology is the possibility of easily designing multispecific proteins which is an attractive characteristic in future combination therapy.

## 5. Conclusions

In summary, the current study demonstrates three important findings which warrant further investigation: (1) there is a functional effect of PD-1 blockade in ovarian cancer-derived TILs, (2) our *ex vivo* stimulation of patient-derived T-cells can be a valuable way to explore the use of PD-1-blocking agents in solid tumors, and importantly, (3) alternative molecular scaffolds with PD-1 antagonizing activity such as DARPin® proteins have the potential to compete with conventional monoclonal antibodies and should be further explored.

## Figures and Tables

**(a):** 

Median age (range)	60 years (41-75)
	Number (*n*) of patients (% of all)
Cancer origin	
Ovarian	11 (64.7%)
Tubal	3^∗^ (17.7%)
Abdominis	2 (11.8%)
Not specified (MB)	1 (5.8%)
FIGO stage	
IIIC	7 (41.2%)
IVA	3 (17.7%)
IVB	5 (29.4%)
IV (no substage specified)	1 (5.8%)
No stage (MB)	1 (5.8%)
Histology	
High-grade serous	14^∗^ (82.4%)
Low-grade serous	1 (5.8%)
Clear cell	1 (5.8%)
MB	1 (5.8%)
Debulking outcome (residual tumor)	
Complete (0 cm)	9 (52.9%)
Optimal (<1 cm)	3 (17.7%)
Incomplete (>2 cm)	5 (29.4%)
Vital status (median follow-up 10 months)	
Alive	11 (64.7%)
Deceased	6 (35.3%)

**(b):** 

Sample type	*n*	Median (range)	CD4/CD8 ratio (range)
Peripheral blood	12	16 (8-27) mL	3.2 (1-6.2)
Ascites	16	738 (217-950) mL	1.0 (0.3-3.6)
Tumor	8	25.1 (2.8-58.0) g	0.6 (0.2-1.6)

Abbreviations: MB: mucinous borderline; FIGO: International Federation of Gynecology and Obstetrics. ^∗^Two patients also had an additional sarcoma component (in addition to tubal origin and high-grade serous histology).

## Data Availability

The data used to support the findings of this study are available from the corresponding author upon reasonable request.

## Supplementary Materials

Supplementary Materials Supplementary Table 1: antibodies used for flow cytometric characterization post-PD-1 blockade. Supplementary Figure 1: PD-L1 expression on CD45- cell fractions from paired ascites and tumor samples (*n* = 6). Supplementary Figure 2: correlation between PD-1 expression and response to *α*-CD3 stimulation. Supplementary Figure 3: assessment of PD-1 binding using Mirrorball laser scanning imaging cytometry. Supplementary Figure 4: associations between PD-1 expression and release of IFN-*γ* after stimulation with *α*-CD3 and PD-1-blocking reagents. Supplementary Figure 5: fold induction of IL-6 from T-cells isolated from ascites (*n* = 2) or tumor tissue (*n* = 4) in the presence of 100 nM of anti-PD-1-directed reagents using a multiplex assay.
