# Supplementary material for: Profound Functional Suppression of Tumor-Infiltrating T-Cells in Ovarian Cancer Patients Can Be Reversed Using PD-1-Blocking Antibodies or DARPin® Proteins
Source: J Immunol Res. 2020 Aug 4;2020:7375947. doi: 10.1155/2020/7375947 (PMC7424497; doi:10.1155/2020/7375947)
Supplement: Supplementary Materials — Supplementary Table 1: antibodies used for flow cytometric characterization post-PD-1 blockade. Supplementary Figure 1: PD-L1 expression on CD45- cell fractions from paired ascites and tumor samples (n = 6). Supplementary Figure 2: correlation between PD-1 expression and response to α-CD3 stimulation. Supplementary Figure 3: assessment of PD-1 binding using Mirrorball laser scanning imaging cytometry. Supplementary Figure 4: associations between PD-1 expression and release of IFN-γ after stimulation with α-CD3 and PD-1-blocking reagents. Supplementary Figure 5: fold induction of IL-6 from T-cells isolated from ascites (n = 2) or tumor tissue (n = 4) in the presence of 100 nM of anti-PD-1-directed reagents using a multiplex assay. [file 7375947.f1.pdf]

# Supplementary information

**Title: Profound functional suppression of tumor-infiltrating T-cells in ovarian cancer patients can be reversed using PD-1 blocking antibodies or DARPin® proteins**

**Authors:** Emelie Foord, Charlotte Klynning, Esther Schoutrop, Judith M. Förster, Jennifer Krieg, Anette Mörtberg, Mischa R. Müller, Christel Herzog, Dieter Schiegg, Denis Villemagne, Ulrike Fiedler, Dan Snell, Benjamin Kebble, Jonas Mattsson, Victor Levitsky, Michael Uhlin

**Supplementary Table I**. Antibodies used for flow cytometric characterization post-PD-1 blockade.

| Target | Fluorochrome | Clone |
|---|---|---|
| CD326/EpCAM | FITC | EBA-1 |
| CD25 | PE | M-A251 |
| CD3 | PE-Cy7 | SK7 |
| CD274/PD-L1 | APC | MIH1 |
| CD8 | APC | RPA-T8 |
| CD45 | Alexa700 | HI30 |
| CD8 | APC-Cy7 | SK1 |
| CD69 | APC-Cy7 | FN50 |
| HLA-DR | APC-H7 | G46-6 |
| CD279/PD-1 | Bv421 | MIH4 |
| CD4 | V500 | RPA-T4 |
| IgG1 isotype ctrl | FITC/APC/Bv421<br>PE | X40<br>MOPC-21 |

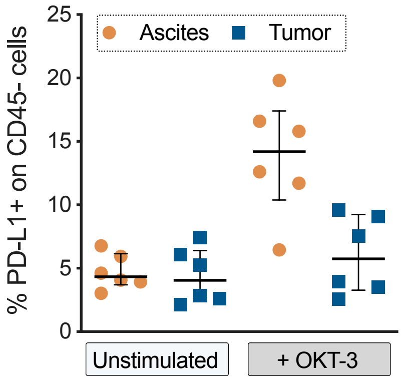


**Supplementary Figure 1. PD-L1 expression on CD45- cell fractions from paired ascites and tumor samples (n=6).** PD-L1 expression was assessed by flow cytometry on unstimulated and stimulated (addition of OKT-3) cell fractions after 48h incubation.

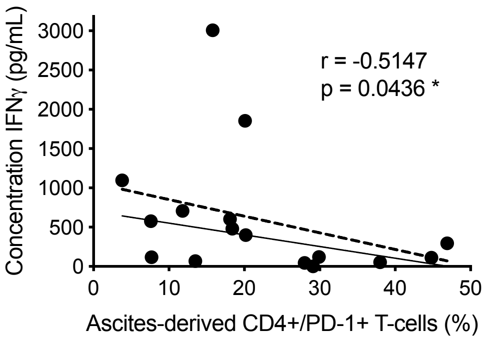


**Supplementary Figure 2. Correlation between PD-1 expression and response to α-CD3 stimulation.** Correlation between PD-1-expressing CD4+ T-cells in ascites (at time of isolation from patient) and the normalized absolute concentration of IFN-γ (per 500 000 T-cells) after stimulation with α-CD3 for 48h. P- and r- values were obtained by Spearman correlation and visualized using non-linear robust regression (solid line) or least squares regression (dashed line). Significance levels were set to $p<0.05$ (*).

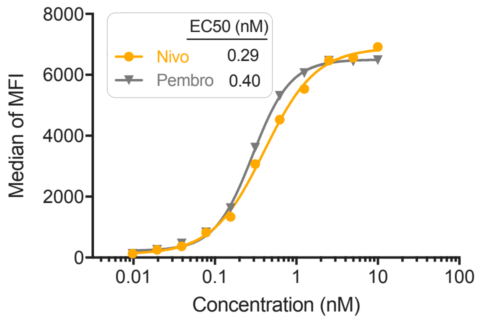


**Supplementary Figure 3. Assessment of PD-1 binding using Mirrorball laser scanning imaging cytometry.** A titration of PD-1-targeting reagents with a PD-1-expressing cell line (transfected HEK293) were used to assess PD-1 binding measured by median of mean fluorescence intensities (MFI). Half effective concentrations (EC50 values) are presented for each PD-1 binding reagent; nivolumab (nivo) in circles, pembrolizumab (pembro) in reversed triangles, DARPin-1 in diamonds and DARPin-2 in squares.

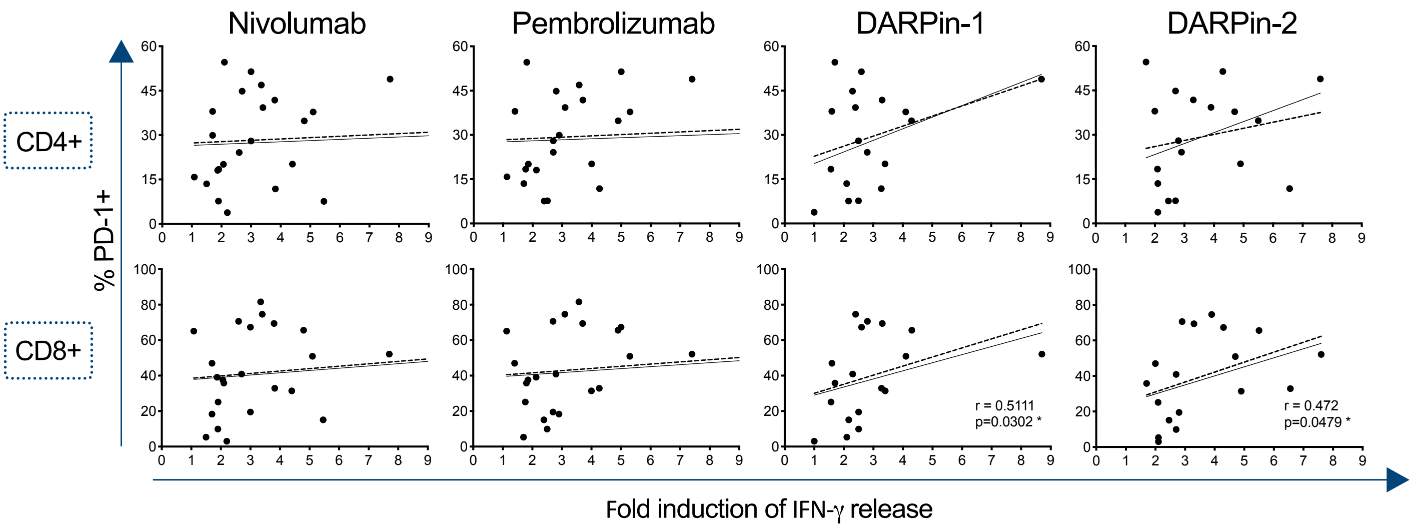


**Supplementary Figure 4. Associations between PD-1 expression and release of IFN-γ after stimulation with α-CD3 and PD-1-blocking reagents.** Correlation analysis for PD-1 expression in CD4+ (upper panel) or CD8+ (lower panel) T-cells isolated from ascites and tumor (from fresh characterization after isolation) and the fold induction of IFN-γ after stimulation with α-CD3 and the four different PD-1 targeting reagents. P- and r- values were obtained by Spearman correlation and dashed lines were plotted using non-linear robust regression (solid line) or least squares regression (dashed line). Significance levels were set to $p<0.05$ (*).

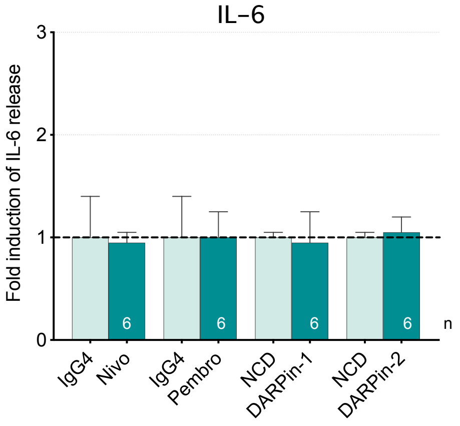


**Supplementary Figure 5. Fold induction of IL-6 from T-cells isolated from ascites (n=2) or tumor tissue (n=4) in presence of 100 nM of α-PD-1-directed reagents using multiplex assay.** Results were generated using 6-plex assay. Fold induction was calculated based on release when adding only α-CD3 with no presence of α-PD-1 reagent or control (represented by dashed line at 1). Four additional ascites samples were initially analyzed but reached the upper detection limit in all conditions (including α-CD3 alone) and were excluded. The number of samples presented is indicated in each bar with presented number of plotted samples presented for each bar (n). Paired Wilcoxon test was used for comparing the response by anti-PD-1 reagent with its corresponding control; IgG4 for mAbs nivolumab (nivo) and pembrolizumab (pembro) or negative control DARPin (NCD) for DARPins. Median values and interquartile ranges are plotted. Significance levels were set to $p<0.05$ (*), $p<0.01$ (**) and $p<0.001$ (***).
